# A Genetic Method for Distinguishing Cryptic *Pocillopora* Species in French Polynesia Without Sequencing

**DOI:** 10.1002/ece3.74409

**Published:** 2026-10-02

**Authors:** Francesca M. Cohn, Erika C. Johnston, Scott C. Burgess, Jordan A. Sims, Krithika Layagala, Pierrick Harnay, Hollie M. Putnam, Adrienne M. S. Correa

**Affiliations:** ^1^ Department of Environmental Science, Policy, and Management University of California Berkeley California USA; ^2^ Department of Developmental Biology Stanford University Stanford California USA; ^3^ Hawaii Institute of Marine Biology University of Hawaii at Manoa Kaneohe Hawaii USA; ^4^ Department of Biological Science Florida State University Tallahassee Florida USA; ^5^ Department of Biology Norwegian University of Science and Technology Trondheim Norway; ^6^ Leon H. Charney School of Marine Sciences University of Haifa Haifa Israel; ^7^ Department of Biological Sciences College of the Environment and Life Sciences, University of Rhode Island Kingston Rhode Island USA

**Keywords:** biodiversity monitoring, coral reef, cryptic species identification, mitochondrial open reading frame (mtORF), *Pocillopora* histone region (PocHistone), restriction fragment length polymorphism (RFLP)

## Abstract

*Pocillopora* is a widespread, dominant reef‐building coral genus in the Indo‐Pacific; many species within this group exhibit high morphological similarity and plasticity. Given this, genetic tools are needed to robustly identify *Pocillopora* individuals to the species level. Quick and accurate identification approaches for *Pocillopora* species are critical to estimating biodiversity patterns under current and future environmental challenges. In recent years, the mitochondrial open reading frame (mtORF) and a histone region (PocHistone) have been validated using genome‐wide data to become the most widely used species‐level markers for *Pocillopora*. However, Sanger sequencing of a large number of samples can be prohibitively expensive and sequencing facilities are not always readily available. Therefore, we present restriction fragment length polymorphism (RFLP) digests here that identify the six species of *Pocillopora* ( *P. acuta* , *P.* cf. *effusa*, *P. grandis* , *P. meandrina* , *P. tuahiniensis*, and *P. verrucosa* ) found in French Polynesia, without sequencing. In uninformed validation tests (in silico and in vitro), our protocol identified each *Pocillopora* species with 100% accuracy. Given their cost‐effective, rapid nature, the tailoring of additional RFLP digest protocols to identify cryptic coral species in reef regions around the world will support foundational reef science, conservation and restoration initiatives.

## Graphical abstract

This study presents restriction fragment length polymorphism (RFLP) digests that identify the six species of *Pocillopora* ( *P. acuta* , *P.* cf. *effusa*, *P. grandis* , *P. meandrina* , *P. tuahiniensis*, and *P. verrucosa* ) found in French Polynesia, without sequencing. In uninformed validation tests (in silico and in vitro), our protocol identified each *Pocillopora* species with 100% accuracy.

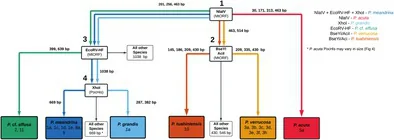

## 1 Introduction

*Pocillopora* (Lamarck, 1816) is a genus of scleractinian reef‐building corals that is widespread across reefs and reef zones in the greater Indo‐Pacific. *Pocillopora* is notoriously plastic in its morphology (Todd 2008; Pinzón et al. 2013; Marti‐Puig et al. 2014; Paz‐García et al. 2015; Gélin et al. 2017; Johnston et al. 2017; Johnston et al. 2018), with environmental factors such as light intensity and water motion (Mass and Genin 2008; Todd 2008; Enríquez et al. 2017; Edmunds and Burgess 2025) contributing to overall skeletal morphology. However, despite gross morphological similarity (Figure 1), cryptic species differ in various ways, such as the corallivore grazing pressure they experience (Burgess et al. 2025), their reproductive timing (Harnay et al. 2025), Symbiodinaceae communities (Johnston, Cunning, and Burgess 2022) and responses to temperature (Burgess et al. 2021) and light (Edmunds and Burgess 2025). These differences contribute to a growing body of evidence that morphologically cryptic *Pocillopora* species do not necessarily share equivalent ecological niches in the reef system (Edmunds and Burgess 2025; Johnston, Cunning, and Burgess 2022; Johnston, Wyatt, et al. 2022). Furthermore, interspecific differences in bleaching support the value of *Pocillopora* as a model for cryptic species resistance and resilience (natural and with human intervention) to climate change (Burgess et al. 2021, 2025; Hughes et al. 2023). The ability to quickly and accurately identify cryptic *Pocillopora* species within a given morphotype (Oury et al. 2023, 2025; Voolstra et al. 2023) is therefore critical to estimating reef biodiversity and understanding how communities of morphologically indistinguishable species may contribute to the robustness of reefs against future environmental challenges (e.g., through portfolio effects, Burgess et al. 2024, Edmunds and Burgess 2025).

**FIGURE 1:**
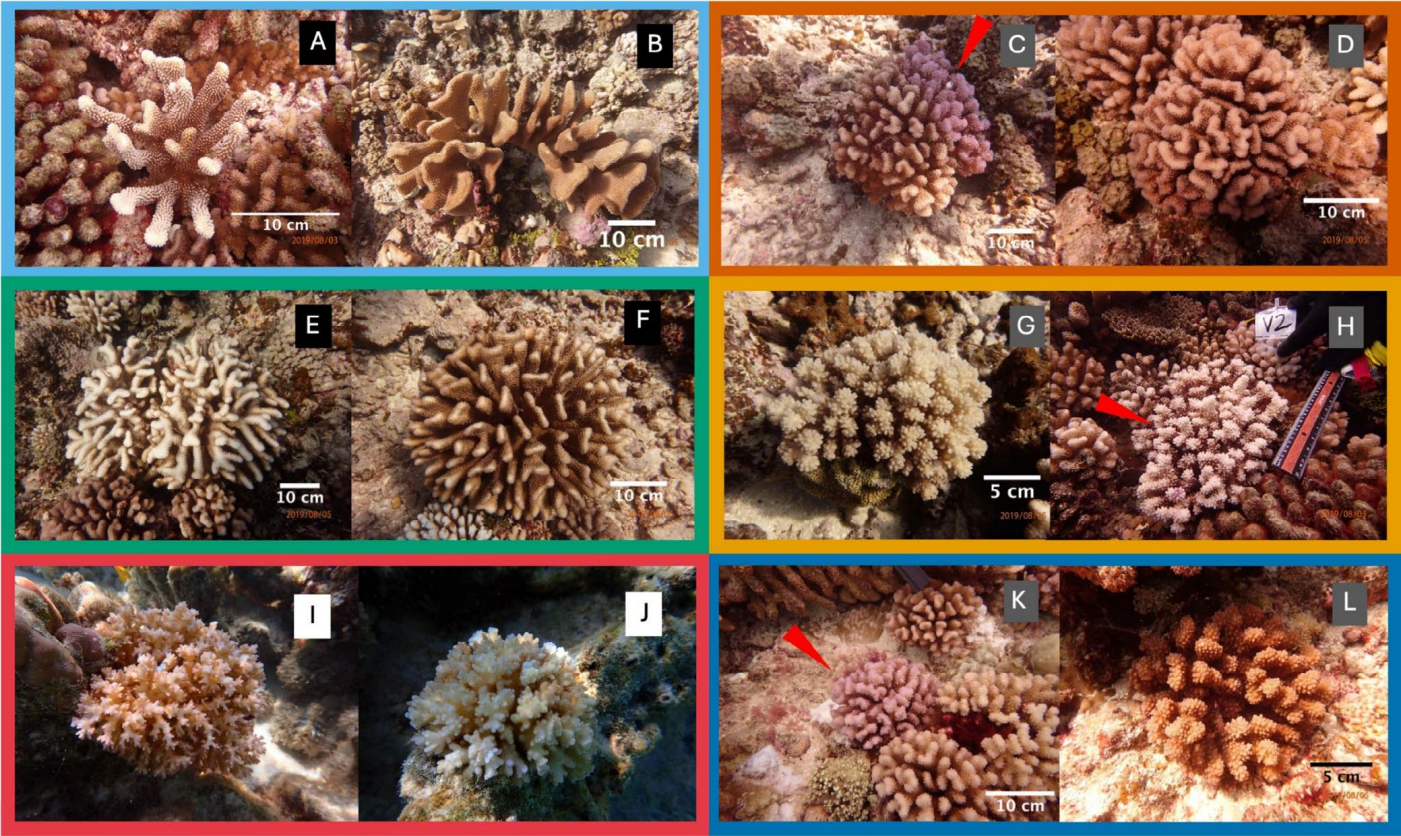
Representative images (A–L) of cryptic *Pocillopora* exhibiting morphological variation within species from Mo'orea, French Polynesia. In images containing multiple colonies, the colony being identified is indicated with a red arrowhead. Despite morphological differences in A and B, both corals are *P. grandis* . Similarly, corals C and D are both *P. tuahiniensis*; E and F are *P*. cf. *effusa*; G and H are *P. verrucosa* ; I and J are *P. acuta* ; and K and L are *P. meandrina* . Note that individuals of different species can look very similar to each other (i.e., *P. tuahiniensis*, C and D; *P. verrucosa* , G and H; *P. meandrina* , K and L) due to phenotypic plasticity in this genus. The boxes behind letters A–L are color coded to group *Pocillopora* spp. that are often confused as adults: *P. grandis* and *P*. cf. *effusa* (black); and *P. tuahiniensis*, *P. verrucosa* , and *P. meandrina* (gray). *P. acuta* (white) is the most reliably identifiable by morphology as an adult. Colored rectangular outlines are used to indicate species and correspond with colors in Figures 3 and 4 (light blue: *P. grandis* ; orange: *P. tuahiniensis*; green: *P*. cf. *effusa*; yellow: *P. verrucosa* ; red: *P. acuta* ; dark blue: *P. meandrina* ). Photos: S.C. Burgess.

To date, approaches using genetic markers (Flot et al. 2008; Pinzón and LaJeunesse 2011; Pinzón et al. 2013; Marti‐Puig et al. 2014; Schmidt‐Roach et al. 2014; Gélin et al. 2017) have delineated species within *Pocillopora*, resolving phylogenetic relationships between historically confused types (Table 1; Johnston, Cunning, and Burgess 2022; Oury et al. 2023). A standardized barcoding method using the mitochondrial open reading frame (mtORF; Flot et al. 2008; Pinzón et al. 2013; Johnston et al. 2018) and a histone region (PocHistone; Johnston et al. 2018) to delineate *Pocillopora meandrina* and *Pocillopora grandis*, which share mtORF haplotype 1, have in recent years become the most widely used species‐level markers for *Pocillopora* (Table 1). Importantly, multiple independent genomic datasets and phylogenetic analyses have organized each mtORF haplotype and PocHistone variant into validated species, allowing researchers to identify species based on these markers, rather than whole genome sequencing (Johnston, Cunning, and Burgess 2022; Oury et al. 2023; Voolstra et al. 2023). However, even Sanger sequencing the mtORF region may be prohibitively expensive for studies involving large sample sizes (Johnston et al. 2018), and sequencing is currently infeasible at many reef locations. A restriction fragment length polymorphism (RFLP) digest and gel protocol was therefore developed to ameliorate these issues in Hawai‘i (Johnston et al. 2018); this protocol supported reliable identification of *Pocillopora* spp. by leveraging interspecific nucleotide differences of the mtORF (Flot et al. 2008) and PocHistone regions (Johnston et al. 2018) without sequencing. Johnston et al. (2018) were the first to uniquely identify *P. meandrina* and *P. grandis* using a histone region.

**TABLE 1:** Table of naming conventions for *Pocillopora* used in prior studies, including mtORF haplotype IDs and lineage names, for the species of *Pocillopora* found in and around Mo'orea, French Polynesia.

mtORF haplotype ID		Corresponding lineages
Pinzón et al. (2013), Forsman et al. (2013), Burgess et al. (2021)	Current lineage name (used in this study)	Greek letters refer to Schmidt‐Roach et al. (2014); Roman numerals refer to Marti‐Puig et al. (2014); Genomic species hypotheses (GSH) refer to Oury et al. (2023)
2, 11	*P*. cf. *effusa*	IIIa, GSH01
3a, 3b, 3c, 3d, 3e, 3f, 3h	*P. verrucosa*	γ, IIa, GSH13a, GSH13c, GSH13b
10	*P. tuahiniensis*	GSH14
5a	*P. acuta*	β, Ia, GSH05
1a, 1c, 1d, 1e, 8a, 9	*P. meandrina*	m, IIb, GSH09b
1a	*P. grandis*	ε, IIb, GSH09c

The RFLP protocol developed by Johnston et al. (2018) was designed to differentiate *Pocillopora* species in Hawai‘i (*Pocillopora acuta*, *Pocillopora damicornis* , *P. grandis* , *Pocillopora ligulata*, *P. meandrina* , and *Pocillopora verrucosa* ), but the complete protocol has not been validated for *Pocillopora* spp. in other geographic regions, which often harbor additional *Pocillopora* haplotypes and species (Oury et al. 2025; Connelly et al. 2026). Several enzymes that were formerly identified as sufficient to differentiate *Pocillopora* in Hawaiʻi are confounded by *de novo* mtORF haplotypes present at other locations due to the presence of different species. For example, the restriction enzyme *SacI‐HF* does not cut haplotype 8a, a haplotype not present in Hawai‘i, but which is a variant of *P. meandrina* in French Polynesia (Johnston et al. 2018; Johnston, Cunning, and Burgess 2022). Additionally, in *Pocillopora* samples from Hawai‘i (Johnston et al. 2018), the use of the restriction enzyme *AlwNI* differentiates *P. ligulata* (haplotype 6) from all other *Pocillopora* species, but this enzyme also cuts mtORF haplotype 11 (*Pocillopora* cf. *effusa*), which is not present in Hawai‘i (Forsman et al. 2013; Gélin et al. 2017). However, in French Polynesia, *AlwNI* cannot be used to differentiate *P*. cf. *effusa* because *P*. cf. *effusa* consists of two mtORF haplotypes, haplotypes 2 and 11 (Johnston, Cunning, and Burgess 2022), and *AlwNI* will not cut mtORF haplotype 2. RFLP protocols that accurately discern cryptic *Pocillopora* species in other regions of the Pacific (besides Hawai‘i; Johnston et al. 2018) are not yet available; extensive knowledge of existing *Pocillopora* species in a given region is a prerequisite for RFLP protocol development.

Here, we describe an RFLP protocol that uses previously established mtORF and PocHistone regions to identify species of *Pocillopora* in French Polynesia, broadening the geographic applicability of RFLP methods for cryptic species identification. The diversity of *Pocillopora* species on Mo'orean reefs has been extensively documented (Forsman et al. 2013; Johnston, Cunning, and Burgess 2022; Johnston, Wyatt, et al. 2022; Burgess et al. 2024), and therefore RFLP methods could be developed for regionally specific mtORF haplotypes and species (e.g., *P*. cf. *effusa*: haplotypes 2, 11; *P. meandrina* haplotype 8a; *P. tuahiniensis*: haplotype 10). Additionally, *Pocillopora* are a dominant reef building genus in this region (Burgess et al. 2021, 2024, 2025; Johnston, Wyatt, et al. 2022; Edmunds and Burgess 2025) contributing to ecological recovery (Tsounis and Edmunds 2016; Burgess et al. 2021) following disturbance events including bleaching events, cyclones, and crown of thorns sea star outbreaks (Pratchett et al. 2011, 2013; Edmunds et al. 2016). Much research to date, however, has documented trends at the genus level (e.g., Pratchett et al. 2013; Speare et al. 2025) because species identification of *Pocillopora* using morphology is unreliable. We anticipate that the relatively inexpensive, rapid and sequencing‐independent protocol presented here for the identification of cryptic *Pocillopora* species will facilitate species level monitoring and research efforts on French Polynesian reefs.

## 2 Methods

Mo'orean reefs harbor broadly distributed, common *Pocillopora* species, as well as several endemic mtORF haplotypes (following the delineation and nomenclature established by Forsman et al. 2013; Pinzón et al. 2013). There are six species, containing 17 associated mtORF haplotypes, that are common on reefs around Mo'orea (Edmunds et al. 2016; Burgess et al. 2021, 2024, 2025; Johnston, Cunning, and Burgess 2022; Johnston and Burgess 2023): *P. acuta* : 5a (Rouzé et al. 2019; Burgess et al. 2024); *P*. cf. *effusa*: 2, 11 (Burgess et al. 2021); *P. grandis* : 1a (Johnston, Cunning, and Burgess 2022; Johnston, Wyatt, et al. 2022; Burgess et al. 2025); *P. meandrina* : 1a (same mtORF as *P. grandis* , but different PocHis amplicon), 1c, 1d, 1e, 8a, 9 (Burgess et al. 2021); *P. tuahiniensis*: 10 (Johnston, Cunning, and Burgess 2022; Johnston and Burgess 2023; Burgess et al. 2025); and *P. verrucosa* : 3a, 3b, 3c, 3d, 3e, 3f, 3h (Table 1; Burgess et al. 2025). *Pocillopora damicornis* and *Pocillopora woodjonesi* have also been reported at low abundances based on gross morphology in the Society Islands (Edmunds et al. 2016), and *Pocillopora damicornis* was reported at low levels in the Southern and Cook Islands based on the mitochondrial ATP synthase subunit 6 (*atp6*) and putative control region (*pmapcr*, Mayfield et al. 2015). However, given the lack of genetic detection of *P. damicornis* and *P. woodjonesi* using widely applied genetic approaches (mtORF and PocHistone) despite extensive studies in Mo'orea (Burgess et al. 2021, 2024, 2025; Johnston et al. 2017; Johnston, Wyatt, et al. 2022; Harnay et al. 2025), these putative species were not included in the RFLP protocol presented here.

To develop a RFLP‐based species identification protocol for *Pocillopora* species in Mo'orea and the greater French Polynesian region, we used an iterative process of: (1) identifying species‐defining single nucleotide polymorphisms (SNPs) in the mtORF and PocHistone regions (Johnston, Cunning, and Burgess 2022; Johnston, Wyatt, et al. 2022) and in silico identification of restriction enzymes that target these sites, and (2) testing the utility of candidate restriction enzymes via in vitro digestion of DNA from previously sequenced *Pocillopora* samples. The digestion protocol was optimized based on the ability of its component restriction enzymes to distinguish *Pocillopora* species and haplotypes based on the fragment band sizes they produced. Lastly, in silico and in vitro uninformed validation tests (via temporary redaction of species IDs) were run on sample sets to ensure accuracy and usability of the finalized enzyme protocol.

### 2.1 In Silico SNP and Enzyme Identification Methods

Species‐specific *Pocillopora* single nucleotide polymorphisms (SNPs) were identified from a *Geneious Prime* (v.2024.0.02) alignment of the 17 unique mtORF haplotypes (1a, 1c, 1d, 1e, 2, 3a, 3b, 3c, 3d, 3e, 3f, 3h, 5a, 8a, 9, 10, 11; Data S1) documented from Mo'orea based on ~4500 samples drawn from Burgess et al. (2021), Johnston, Wyatt, et al. (2022), Burgess et al. (2024), S.C. Burgess et al. (unpublished), and P. Harnay et al. (unpublished). These mtORF haplotypes are anchored to type material based on morphometric comparisons and location (Schmidt‐Roach et al. 2014; Johnston and Burgess 2023), except for *Pocillopora effusus* (Glynn et al. 1996; Pinzón et al. 2013), which we therefore refer to as *P*. cf. *effusa* in this study. Candidate restriction enzymes were tested in silico using the *Restriction Analysis* feature of *Geneious Prime*. Restriction enzymes were selected for testing and validation based on their potential to differentiate two or more of the focal *Pocillopora* species. A similar selection process was attempted using an alignment of the PocHistone amplicon for haplotypes associated with each focal *Pocillopora* species based on a total of 41 samples (Johnston et al. 2018; Data S2). However, high intra‐haplotype variability in the PocHistone amplicon limited this approach as exemplified by the attempted *HgaI* digest of PocHistone described below (Figure S1). Therefore, the PocHistone amplicons were only used as previously described by Johnston et al. (2018) in a *XhoI* digest to differentiate *P. grandis* and *P. meandrina* . To determine the location of each enzyme's cut sites and all expected band sizes of the cut mtORF amplicon, the Flot et al. (2008) primers were mapped onto the *P. grandis* mitochondrial genome (GenBank accession NC_009798.1). This reference genome was added to the mtORF alignment to determine where predicted cut sites fell within the mtORF amplicon.

### 2.2 In Vitro Enzyme Testing Methods

A total of eight candidate enzymes were identified for further testing in silico: *AciI* (New England Biolabs (NEB), MA, USA, #R0551S), *BanI* (NEB, #R0118S), *BceAI* (NEB, #R0623S), *BseYI* (NEB, #R0635S), *EcoRV‐HF* (NEB, #R3195S), *HgaI* (NEB, #R0154S), *NlaIV* (NEB, #R0126S), *SacI‐HF* (NEB, #R3156S), and *XhoI* (NEB, #R0146S). Each enzyme was used in in vitro restriction digests applied to mtORF amplicons generated from 42 samples of *Pocillopora* species representing 12 haplotypes (1a, 2, 3a, 3b, 3e, 3f, 3h, 5a, 8a, 10, 9, 11), collected from 5, 10, and 20 m depth at four sites across the fringing, back, and forereef zones of Mo'orea (Burgess et al. 2021; Johnston, Wyatt, et al. 2022; Burgess et al. 2024, S.C. Burgess et al. unpublished). Additionally, the associated PocHistone amplicon of each of the above samples was tested with *XhoI* to test the digest's ability to differentiate *P. grandis* and *P. meandrina* species among haplotypes from French Polynesia. The utility of restriction enzyme digests in accurately parsing *Pocillopora* species was assessed based on: (1) the extent to which the RFLP ‘fingerprints’ produced by an enzyme matched the band size expected based on in silico analysis of sequence data; and (2) the ability of human users to accurately visually distinguish distinct banding patterns in gels.

In preparation for restriction enzyme digest tests, 50 μL aliquots of mtORF amplicons were generated from DNA extractions of the 42 *Pocillopora* samples using primers FatP6.1 (5′‐TTTGGGSATTCGTTTAGCAG‐3′) and RORF (5′‐SCCAATATGTTAAACASCATGTCA‐3′; Flot et al. 2008). Polymerase chain reactions (PCRs) were run using Taq PCR Master Mix Kits (Qiagen, Hilden, Germany) and contained 25 μL of master mix, 22 μL of H_2_O, 1 μL of each primer (forward and reverse, 10 μM), and 1 μL of sample DNA (~20–30 ng/μL). The thermocycler protocol included initial denaturation at 94°C for 60 s; 40 cycles of 94°C for 30 s, 53°C for 30 s, and 72°C for 75 s; and a final extension of 72°C for 5 min (Flot et al. 2008; Johnston et al. 2018). PocHistone amplicons were also generated from DNA from each *Pocillopora* sample using PocHistoneF (5′‐ATTCAGTCTCACTCACTCACTCAC‐3′) and PocHistoneR primers (5′‐TATCTTCGAACAGACCCACCAAAT‐3′; Johnston et al. 2018) using the same reagents and reaction volumes detailed above for mtORF reactions. However, the following thermocycler program was used in PocHistone PCR reactions: initial denaturation at 94°C for 60 s; 40 cycles of 94°C for 30 s, 53°C for 30 s, and 72°C for 1 min; and a final extension of 72°C for 5 min. Amplicons were stored at −20°C prior to undergoing restriction enzyme digestion.

### 2.3 Restriction Enzyme Digestion Conditions for In Vitro Testing

To perform enzyme digestions, 5 μL of PCR amplicons generated from each *Pocillopora* species were digested with 0.5 μL of digestion buffer and 0.5 μL of one restriction enzyme or 1 μL of two combined restriction enzymes for 1 h at 37°C, followed by a 20‐min enzyme denaturation step. Reaction reagents and denaturation temperatures varied for each restriction enzyme assay and are detailed in Table 2. Following digestion, 5 μL of digested PCR amplicons were run on a 2% agarose gel at 100 V for 45 min. Length of DNA fragments produced was determined by comparison to a 50 bp DNA ladder (NEB, #N3236). Gels were stained with Invitrogen SYBR Safe DNA gel stain (ThermoFisher Scientific, MA, USA) and visualized on a GelDoc Go imaging system (Bio‐Rad, CA, USA). The restriction enzyme *NlaIV* was previously validated for grouping *P. damicornis* and *P. acuta* in a RFLP protocol for Hawai‘i (Johnston et al. 2018) and was not included in the in vitro enzyme identification process; this enzyme was included, however, in the in vitro uninformed validation.

**TABLE 2:** Amplicon and reagent volumes, and restriction enzyme denaturation temperatures for all restriction enzyme digestions tested. All restriction enzyme digests were run at 37°C for 1 h prior to denaturation.

Digestion name	Amplicon volume	Restriction enzyme volume	Digestion buffer volume	Denaturation temperature
AciI/BseYI	5 μL mtORF	0.5 μL *AciI* 0.5 μL *BseYI*	0.5 μL NEBuffer r3.1	80°C
BceAI/BanI	5 μL mtORF	0.5 μL *BceAI* 0.5 μL *BanI*	0.5 μL rCutSmart Buffer	65°C
EcoRV‐HF	5 μL mtORF	0.5 μL *EcoRV‐HF*	0.5 μL rCutSmart Buffer	65°C
HgaI	5 μL mtORF	0.5 μL *HgaI*	0.5 μL rCutSmart Buffer	65°C
NlaIV	5 μL mtORF	0.5 μL *NlaIV*	0.5 μL rCutSmart Buffer	65°C
SacI‐HF/EcoRV‐HF	5 μL mtORF	0.5 μL *SacI‐HF* 0.5 μL *EcoRV‐HF*	0.5 μL rCutSmart Buffer	65°C
XhoI	5 μL PocHistone	0.5 μL *XhoI*	0.5 μL rCutSmart Buffer	65°C

### 2.4 In Silico and In Vitro Uninformed Validation Methods

In silico uninformed validations of *BseYI/AciI*, *EcoRV‐HF*, and *NlaIV* were next performed on an mtORF haplotype dataset generated from 673 *Pocillopora* samples collected in French Polynesia (Johnston, Wyatt, et al. 2022), a subset of the ~4500 samples used to construct the alignment used for the In Silico *SNP and Enzyme Identification Methods*. Using the *Restriction Analysis* feature of Geneious Prime 2024.0.02, mtORF haplotypes were digested with *BseYI*/*AciI*, and each haplotype was categorized as either *P. verrucosa* , *P. tuahiniensis*, or one of the other four *Pocillopora* species. A *Restriction Analysis* digest was then conducted with *EcoRV‐HF*, and each haplotype was categorized as either *P*. cf. *effusa* or one of the other five *Pocillopora* species. A final *Restriction Analysis* digest was then run with *NlaIV*, and each haplotype was categorized as (1) *P. tuahiniensis* or *P. verrucosa* , (2) *P. grandis* , *P. meandrina* , or *P*. cf. *effusa*, or (3) *P. acuta* . The predicted species for each sample was then compared against the sample metadata to confirm that the correct species had been identified by the in silico analyses. *XhoI* was not included in the uninformed test because PocHistone sequences were not available from Johnston, Wyatt, et al. (2022). However, *XhoI* cutting of PocHistone amplicons was extensively shown to distinguish *P. grandis* and *P. meandrina* by Johnston et al. (2018) and it was established that *XhoI* digested the *P. grandis* haplotype, while leaving all *P. meandrina* haplotypes uncut (including the endemic 8a haplotype) during in silico enzyme testing.

An in vitro uninformed validation was then conducted on 36 samples of *Pocillopora*, representing eight haplotypes from *P. acuta* (haplotype 5a; *n* = 5 from Mo'orea and *n* = 1 from University of Rhode Island (URI) aquarium), *P. meandrina* (haplotypes 1a, 8a; *n* = 5 each), *P*. cf. *effusa* (haplotype 11; *n* = 5), *P. grandis* (haplotype 1a; *n* = 5), *P. tuahiniensis* (haplotype 10; *n* = 5), and *P. verrucosa* (haplotypes 3a, *n* = 1; 3b, *n* = 4) sampled at 2 m depth from the lagoonal reefs of the north shore of Mo'orea (Figure S2, Table S1). Initial species identifications were performed at URI by Sanger sequencing the mtORF region using FatP6.1 and RORF primers (Data S3). Samples with haplotype 1 were then amplified using PocHistone primers, digested with *XhoI*, and visualized as described above for species‐level identification. DNA aliquots of each sample (with species IDs redacted) were sent to the University of California, Berkeley, where the uninformed validation of this protocol was performed. PocHistone and mtORF amplicons were generated and each of the four digests plus an additional alternative *BceAI/BanI* digest (Materials and Methods S1: *BanI/BceAI Digest*) were run according to the methods described in *In Vitro Enzyme Identification*.

## 3 Results

### 3.1 In Silico SNP and Enzyme Identification

Here, we detail restriction enzyme cut sites used, based on the presence of fixed SNPs that differentiate *Pocillopora* species. Note that because some of the enzymes in this protocol have staggered cut sites (e.g., *BseYI*), the positions described here are calculated from the 5′ forward primer (FatP6.1 or forward PocHistone) to the 5′‐most cut site on the top strand. The top strand is then used to identify the position of all subsequent cut sites on each haplotype. *P. acuta* (haplotype 5a) was differentiated from all other *Pocillopora* species in Mo'orea via mtORF using a previously identified fixed Guanine SNP at position 341 recognized by *NlaIV* and validated in Johnston et al. (2018). This polymorphism additionally distinguished *P. meandrina* , *P. grandis* , and *P*. cf. *effusa* from all other species for further differentiation by a fixed guanine SNP at position 312 in *P*. cf. *effusa*, *P. acuta* , *P. meandrina* , and *P. grandis* . All species share a cut site for this restriction enzyme at positions 514 and 515.

To differentiate *P*. cf. *effusa* (haplotype 2, 11) from all other species, a fixed cytosine SNP at position 342 was identified as a cut site of the restriction enzyme *EcoRV‐HF* in the multispecies mtORF alignment. This fixed cytosine SNP at 342 bp in *P*. cf. *effusa* is not shared with any other species.

*Pocillopora verrucosa* (haplotypes 3a, 3b, 3c, 3d, 3e, 3f, 3h) and *P. tuahiniensis* (haplotype 10) were differentiated from each other and all other species using a co‐digest of *AciI* and *BseYI* targeting SNPs in the mtORF alignment. To differentiate *P. verrucosa* from most species, a fixed cytosine SNP at position 210 was identified in *P. verrucosa* and *P. tuahiniensis* and is recognized by *AciI*. The same restriction cut site is shared by all species at position 546. To simultaneously differentiate *P. tuahiniensis* from all other species, a fixed cytosine SNP is used at position 398 and recognized by *BseYI*.

*Pocillopora grandis* and *P. meandrina* are easily confused at small colony sizes and share the 1a mtORF haplotype. The Johnston et al. (2018) protocol uses a *XhoI* digest of the PocHistone amplicon to differentiate these species, as *XhoI* cuts *P. grandis* , but not *P. meandrina*, due to a fixed thymine polymorphism at position 279. In the process of designing this protocol, it was identifi ed. that the *XhoI* digest of *P. grandis* amplicons produces either two or three bands when run out on a gel. Further sequencing (restriction site associated DNA sequencing, RADseq) of *Pocillopora* samples from Mo'orea has revealed that some *P. grandis* individuals are heterozygotic for the *XhoI* restriction cut site (5′C^TCGAG3′), suggesting that some individuals may be monozygotic for absence of the cut site. Of 194 samples collected from Mo'orea that were identified as *P. meandrina* by sequencing the mtORF region followed by the PocHistone RFLP protocol (i.e., these samples were monozygotic for absence of the *XhoI* cut site), only one sample out of 194 (0.5% of colonies analyzed), was erroneously identified as *P. grandis* following the analysis of RADseq data (Materials and Methods S1: *RADseq Histone Analysis*).

### 3.2 In Vitro Enzyme Testing

*EcoRV‐HF* (mtORF), *BseYI/AciI* (mtORF), *NlaIV* (mtORF), and *XhoI* (PocHistone) were selected for this protocol because they produced RFLP ‘fingerprints’ that matched the band sizes expected based on in silico analysis of sequence data and were easily distinguishable visually on gels (Table 3, Figure 2). Other enzymes, such as *SacI‐HF* and *HgaI*, were not selected for the protocol because haplotypes of a given *Pocillopora* species exhibited different banding patterns when digested (Figures S1, S3). While other enzymes were possible candidates for the final protocol, including *BceAI/BanI* to identify *P*. cf. *effusa* and group together *P. grandis* (Haplotype 1), *P. meandrina* (Haplotype 1a, 1c, 1d, 1e, 8a, 9), and *P. acuta* (Haplotype 5a), these were ultimately not selected for the protocol because they increased the number of enzymes required to identify all *Pocillopora* species sequentially (Materials and Methods S1: *BanI/BceAI Digest*, Figure S4, Table S2).

**TABLE 3:** Summary of *Pocillopora* species found in Mo'orea, French Polynesia, their associated haplotype IDs, the amplicon(s) and restriction enzyme(s) used for identification, the resulting fragment sizes for the focal species, and the resulting fragment size(s) for non‐focal species when cut with that restriction enzyme. Corresponding information regarding additional digests required (if any) to fully identify each *Pocillopora* species are provided. Note that the 30 bp fragment resulting from cutting mtORF amplicons of *P. acuta* with *NlaIV* is too small to be reliably observed on a 2% agarose gel; instead a band pattern of 172, 313, and 463 bp fragments should be visible for this species (see Figure 2).

Focal *Pocillopora* species	Haplotype IDs	Amplicon	Restriction enyzme	Resulting fragment size(s) for focal species (bp)	Resulting fragment size(s) for non‐focal species (bp)	Additional mtORF digests required to ID focal species?	Additional amplicon?	Restriction enyzme	Resulting fragment size(s) for focal species (bp)	Resulting fragment sizes for non‐focal species (bp)
*P. verrucosa*	3a, 3b, 3c, 3d, 3e, 3f, 3h	mtORF	BseYI C'CCAGC GGGTC'G AciI C'CGC GGC'G	209, 337, 432	*P. tuahiniensis*: 149, 188, 209, 432; All other species: 432, 546	No	No	N/a	N/a	N/a
*P. tuahiniensis*	10	mtORF	BseYI C'CCAGC GGGTC'G AciI C'CGC GGC'G	149, 188, 209, 432	*P. verrucosa* : 209, 337, 432; All other species: 432, 546	No	No	N/a	N/a	N/a
*P*. cf. *effusa*	2, 11	mtORF	EcoRV‐HF GAT'ATC CTA'TAG	339, 639	All other species: 978	No	No	N/a	N/a	N/a
*P. acuta*	5a	mtORF	NlaIV GGN'NCC CCN'NGG	30, 172, 313, 463	*P. meandrina* , *P. grandis* , *P*. cf. *effusa*: 202, 313, 463; *P. verrucosa* , *P*. *tuahiniensis*: 463, 515	No	No	N/a	N/a	N/a
*P. meandrina*	1a, 1c, 1d, 1e, 8a, 9	mtORF	NlaIV GGN'NCC CCN'NGG	202, 313, 463	*P. acuta*: 30, 172, 313, 463; *P. verrucosa* , *P. tuahiniensis*: 463, 515; *P*. cf. *effusa*: 202, 313, 463	EcoRV‐HF to distinguish from *P*. cf. *effusa*	PocHistone	XhoI C'TCGAG GAGCT'C	669 (Johnston et al. 2018)	*P. grandis* : 287, 382; All other species: 669
*P. grandis*	1a	mtORF	NlaIV GGN'NCC CCN'NGG	202, 313, 463	*P. acuta* : 30, 172, 313, 463; *P. verrucosa* , *P*. *tuahiniensis*: 463, 515; *P*. cf. *effusa*: 202, 313, 463	EcoRV‐HF to distinguish from *P*. cf. *effusa*	PocHistone	XhoI C'TCGAG GAGCT'C	287, 382 (Johnston et al. 2018)	All other species: 669

**FIGURE 2:**
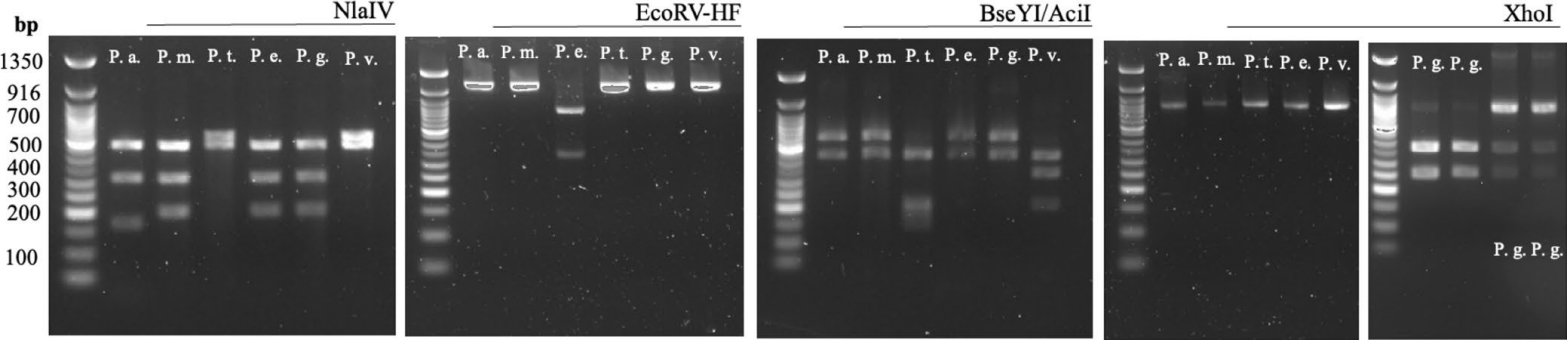
Gel band patterns generated by each restriction enzyme digest, which together, identify species of *Pocillopora* found in French Polynesia: *Pocillopora acuta* (P.a.), *Pocillopora meandrina* (P.m.), *Pocillopora tuahiniensis* (P.t.), *Pocillopora* cf. *effusa* (P.e.)., *Pocillopora grandis* (P.g.), *Pocillopora verrucosa* (P.v.), Left to right: *NlaIV* digest of the mtORF amplicon for all six *Pocillopora* species (The 30 bp fragment for *P. acuta* mtORF amplicons cut with *NlaIV* is too small to be reliably observed; a band pattern of 172, 313, and 463 bp fragments should be visible for this species, see Table 3); *EcoRV‐HF* digest of the mtORF amplicon for all six *Pocillopora* species; *BseYI/AciI* digest of the mtORF amplicon for all six *Pocillopora* species; *XhoI* digest of the PocHistone amplicon for all six *Pocillopora* species. The *P. grandis* digests for *XhoI* are four different samples, representative of the (left to right) two‐band vs. three‐band cut patterns.

The *EcoRV‐HF* (mtORF), *BseYI/AciI* (mtORF), *NlaIV* (mtORF), and *XhoI* (PocHistone) RFLP digests are combinatorially able to unambiguously identify the six *Pocillopora* species present in Mo'orea. For the majority of *Pocillopora* samples, only one digest was needed to identify the corresponding species after the sample had been amplified at the mtORF or PocHistone regions. The *EcoRV‐HF* digest identified *P*. cf. *effusa* (mtORF); the *NlaIV* digest identified *P. acuta* (mtORF); the *BseYI/AciI* digest identified each of *P. tuahiniensis* and *P. verrucosa* , respectively (mtORF); the *XhoI* digest identified *P. grandis* (PocHistone). Three digests and two markers are required to identify *P. meandrina* : *NlaIV* (mtORF), *EcoRV‐HF* (mtORF), and *XhoI* (PocHistone) (Table 3). Using these enzymes, an unknown *Pocillopora* sample from French Polynesia can be identified to species using a sequential protocol of up to six steps (*Finalized Sequential Protocol*).

### 3.3 In Silico and In Vitro Uninformed Validation of the Protocol

The in silico uninformed validation of *BseYI/AciI*, *EcoRV‐HF*, and *NlaIV* digestions identified 100% of the samples (*n* = 673) accurately based on each digest. Additionally, the in vitro uninformed validation identified 100% of samples (*n* = 36) to species accurately by RFLP, as confirmed by prior (uninformed) sequencing of the mtORF region of each sample (Figure 3, Data S3).

**FIGURE 3:**
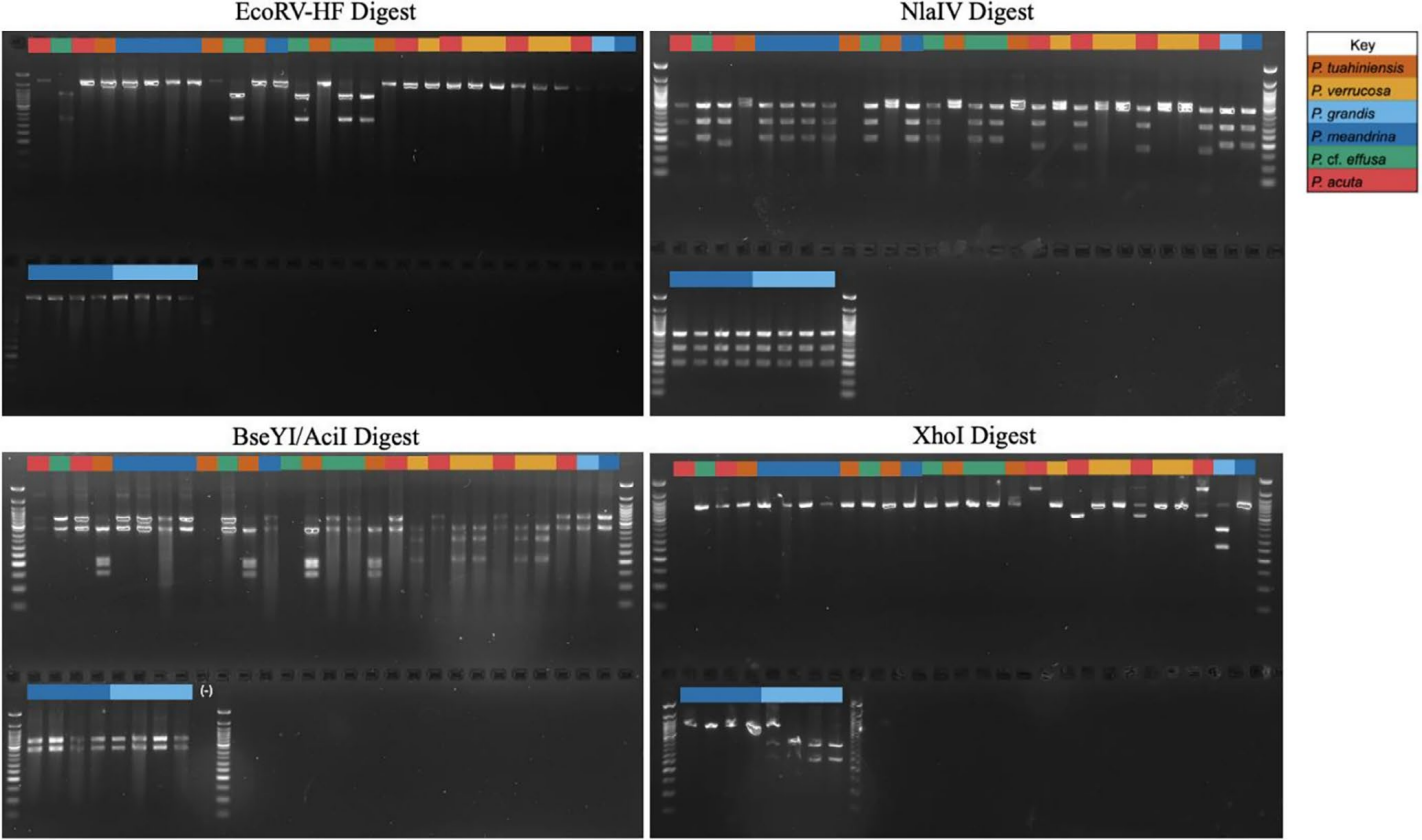
Results of the in vitro uninformed validation for each digest run on 36 unidentified *Pocillopora* samples representing *P. acuta* , *P*. cf. *effusa*, *P. grandis* , *P. meandrina* , *P. tuahiniensis*, and *P. verrucosa* collected in French Polynesia. Lanes 1, 9, and 13 did not appear in some digests but were still identifiable from running all four digests. All samples were identified accurately. See Figures S5 and S6 for additional information on amplicon and fragment size variation in the *XhoI* Digest.

### 3.4 Finalized Sequential Protocol

For a collection of unidentified *Pocillopora* samples, the sequential protocol is (Figure 4):

1. PCR amplify the mtORF region of each sample using the FatP6.1 and RORF primers.
2. Digest the mtORF amplicons with the restriction enzyme *NlaIV* to (1) distinguish *P. acuta* from all other species, (2) group *P. tuahiniensis*/ *P. verrucosa* , and (3) group *P*. cf. *effusa*/ *P. meandrina* / *P. grandis* .
3. For samples that are in the *P. tuahiniensis*/ *P. verrucosa* group, digest the mtORF amplicons with the restriction enzymes *BseYI* and *AciI* to distinguish *P. tuahiniensis* and *P. verrucosa* .
4. For samples that are in the *P*. cf. *effusa*/ *P. meandrina* / *P. grandis* group, digest the mtORF amplicons with the restriction enzyme *EcoRV‐HF* to distinguish *P*. cf. *effusa* from *P. meandrina* / *P. grandis* .
5. For samples that are in the *P. meandrina* / *P. grandis* group, PCR amplify the PocHistone amplicon using the PocHistoneF and PocHistoneR primers.
6. Digest the PocHistone amplicons with the restriction enzyme *XhoI* to distinguish *P. meandrina* and *P. grandis* .

**FIGURE 4:**
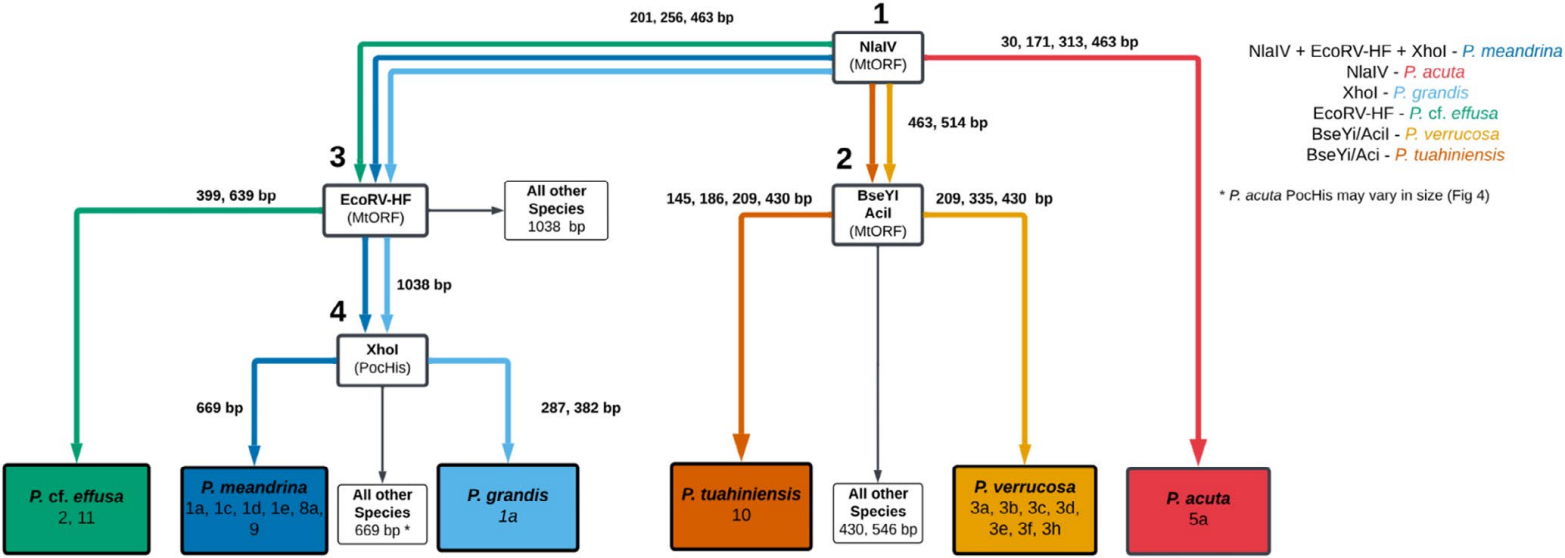
Flow chart of restriction enzyme digests required for the identification of each species of *Pocillopora* in French Polynesia. Colored arrows correspond to the digestion path taken through the diagram for each species. The large numbers indicate the order the digests should be performed in as part of the Finalized Sequential Protocol to identify all species with no prior information.

## 4 Discussion

This study presents a restriction enzyme digest method to rapidly and unambiguously differentiate the six species of cryptic *Pocillopora* present in Mo'orea, French Polynesia, based on their mtORF and PocHistone haplotypes. Taken together with the methods described in Johnston et al. (2018), this study indicates that the development of additional region‐specific, rapid, and sequencing‐free RFLP protocols holds merit for cryptic coral species identification in geographic reef regions (e.g., the wider Pacific and Red Sea for *Pocillopora*).

In Mo'orea, the morphological similarity and plasticity of *Pocillopora* species have historically posed a major roadblock to species‐specific research (Figure 1; Johnston et al. 2017; Johnston, Cunning, and Burgess 2022; Burgess et al. 2024, 2025). For example, *P. tuahiniensis*, *P. verrucosa*, and *P. meandrina* share similar gross skeletal morphologies (Figure 1; Johnston, Wyatt, et al. 2022; Johnston and Burgess 2023; Burgess et al. 2024) and an overlapping depth distribution (2–20 m; Burgess et al. 2024). The method presented here provides a single digest of the mtORF amplicon with *BseYi/AciI* to identify *P. verrucosa* and *P. tuahiniensis*, and distinguish between them (Table 3). *P. meandrina* and *P. grandis* are also often difficult to distinguish at smaller sizes, and both can be confused with *P. verrucosa* (Burgess et al. 2025). *XhoI* digest of the PocHistone amplicon identifies *P. grandis* samples; additional digests of mtORF amplicons with *NlaIV*, *EcoRV‐HF*, and *BseYI/AciI*, respectively, are required to distinguish *P. meandrina* from *P. verrucosa* (Table 3). Finally, adult *P. grandis* and *P*. cf. *effusa* can attain large colony sizes and be morphologically indistinguishable (figure 1; Johnston, Wyatt, et al. 2022). These species can be separated with a single digest of the mtORF amplicon with *EcoRV‐HF* to identify *P*. cf. *effusa* samples, followed by an additional *XhoI* digest of the PocHistone amplicon to identify *P. grandis* (Table 3). As suggested by these examples, the methods presented in this paper can be used individually to identify specific target species, or combined to identify all species, making the protocol highly customizable to the identification needs of any project involving French Polynesian *Pocillopora* species. However, in most cases (especially for coral recruits), the finalized sequential protocol (Figure 4) must be run to accurately identify all *Pocillopora* species by process of elimination.

The biggest advantage of the method presented here is that it can be conducted at many field sites, while eliminating the need for access to sequencing facilities for species‐level identification of *Pocillopora*. Remote reefs, including some reefs of French Polynesia, can present logistical challenges for molecular work and highlight the need for field‐adaptable genetic identification protocols such as the one we present here. With the advent of small, modular, and open‐source PCR technology (e.g., prebuilt kits, DIY arduino or raspberry pi‐based thermocyclers), the methods described in this paper allow for fast and efficient identification of *Pocillopora* species at sites without sequencing facilities (see example protocols at Cohn et al. 2026).

This RFLP protocol provides a cost‐effective alternative to the amplicon Sanger sequencing that was previously required to resolve morphologically similar *Pocillopora* species (Johnston et al. 2018). At the time of publication, the enzymes and associated buffers used in these methods cost approximately $4.20 USD per sample to run all four digests without any enzyme dilutions—roughly two‐thirds the cost of Sanger sequencing ($7.00 USD total to sequence in both directions). Additionally, depending upon the concentration of amplicons, 1:1 enzyme dilutions in water may be used to cut RFLP protocol costs in half (to $2.10 USD per sample); this is roughly one‐third of Sanger sequencing costs. Therefore, the RFLP protocol provides a flexible range of pricing that depends on the enzymes required, as opposed to a fixed sequencing rate that may be unnecessarily costly (i.e., when trying to distinguish between only two species, such as *P. verrucosa* and *P. tuahiniensis*).

We detected variation in the PocHistone amplicon used to differentiate *P. grandis* from *P. meandrina* . Unlike the mtORF amplicon, a mitochondrial marker, the PocHistone amplicons are generated from nuclear DNA and may therefore be heterozygous. We found that *P. grandis* amplicons in French Polynesia produce either two or three bands following digestion, suggesting that some of the population is heterozygous for the *XhoI* cut site (5′ C/TCGAG 3′; Figure 2). While this does not interfere with identification of *P. grandis* using our RFLP protocol, this finding indicates that there may be *P. grandis* colonies that are homozygous for the absence of the *XhoI* cut site. However, variation in this *P. grandis* cut site that leads to the enzyme not recognizing the site (i.e., C/TCGCG, as is observed in *P. meandrina* ) appears to be very rare—of the 194 colonies identified as either *P. meandrina* or *P. grandis* , only one (0.5%) of the *P. grandis* colonies was homozygous for the *XhoI* cut site absence (C/TCGCG) (Materials and Methods S1: *RADseq Histone Analysis*).

We also found a PocHistone amplicon anomaly among *P. acuta* samples. This included a 345 bp indel starting at bp position 538 (identified by Sanger sequencing; GenBank accession PZ381813), resulting in a substantially longer PocHistone amplicon (~1000 bp) compared to the 669 bp observed in other species (Figure S5). Additionally, another *P. acuta* anomaly exists where another band ~500 bp is present, sometimes with the 1014 bp indel band and expected 669 bp band. However, this does not impact the ability to identify *P. grandis* with the *XhoI* digest, as only *P. grandis* presents the diagnostic 287 and 382 bp bands when digested (Figure S6). Despite these caveats, the uninformed validation used in this study was able to identify *Pocillopora* samples (*n* = 36) to species with 100% accuracy (Figure 3).

In terms of mtORF variation, it is noteworthy that in French Polynesia, *P. verrucosa* is represented by a large number of haplotypes (3a, 3b, 3c, 3d, 3e, 3f, 3h), and many of these haplotypes have few SNP differences between them (e.g., 3b and 3d, 3h and 3e: one SNP difference; 3a and 3b: two SNP differences). In Mo'orea, these haplotypes all correspond to *P. verrucosa* (Johnston, Cunning, and Burgess 2022). However, in some study locations outside of French Polynesia, some of these haplotypes represent distinct species. For example, mtORF haplotype 3a and 3d are identified as *Pocillopora lacera* in the Eastern Tropical Pacific (Oury et al. 2023, 2024; Connelly et al. 2026), whereas haplotypes 3c, 3e, 3g, 3h, and 7a are identified as *Pocillopora favosa* in the Arabian Peninsula (Oury et al. 2025).

For *Pocillopora* species beyond Mo'orea, mtORF haplotypes have been characterized at neighboring islands, including Tetiaroa, Tahiti, and Maiao (Edmunds et al. 2016), with no new haplotypes identified at these locations. The Tara Pacific expedition (2016–2018) sampled colonies resembling a typical *P. meandrina* morphology spanning 18,000 km of the Pacific Ocean, including Mo'orea, Tenoko, Tekava, and Kamaka from the Gambiers, and Aitutaki from the Cook Islands. Samples of *Pocillopora* species from the expedition did not identify any haplotypes unaccounted for in our methods, further supporting the applicability of our method to the larger region of French Polynesia. However, we do caveat that study sites located further from Mo'orea have the potential to contain species or haplotypes not represented in our results, which may reduce the accuracy of the RFLP protocol presented here at those locations. For example, *P. damicornis* , identified using variable sites with the mitochondrial ATP synthase subunit 6 (*atp6*) and putative control region (*pmapcr*), has been found in low abundance in the Southern and Cook Islands of French Polynesia and is not included in these methods (Mayfield et al. 2015). However, if applying the RFLP protocol here to the southern islands, it is likely that an additional digest of *Tsp45I* can be added after the *NlaIV* digest to distinguish *P. acuta* from *P. damicornis* as indicated in Johnston et al. (2018), although the mtORF haplotypes of *P. damicornis* in French Polynesia are unconfirmed.

## 5 Conclusion

This paper presents and validates a highly accurate, rapid, and low‐cost sequence‐free method for differentiating the cryptic *Pocillopora* species present in Mo'orea and the broader geography of French Polynesia using mtORF and PocHistone amplicons. We demonstrate that RFLP protocols can be developed to identify cryptic *Pocillopora* species in regions other than the Hawaiian Islands; additional protocols could be tailored for other reef regions in the Pacific and the Red Sea (Oury et al. 2025). As cryptic species are increasingly identified across coral genera (Grupstra et al. 2024; Rassmussen et al. 2025), approaches for RFLP protocol development, such as those presented here, are likely to become increasingly necessary globally.

## Author Contributions

**Francesca M. Cohn:** conceptualization (lead), data curation (equal), formal analysis (lead), investigation (lead), methodology (lead), validation (equal), visualization (lead), writing – original draft (lead), writing – review and editing (equal). **Erika C. Johnston:** conceptualization (supporting), data curation (supporting), formal analysis (equal), methodology (equal), resources (equal), validation (equal), visualization (supporting), writing – original draft (supporting), writing – review and editing (equal). **Scott C. Burgess:** conceptualization (equal), data curation (supporting), formal analysis (equal), funding acquisition (equal), investigation (supporting), methodology (supporting), resources (lead), validation (equal), writing – original draft (supporting), writing – review and editing (equal). **Jordan A. Sims:** investigation (supporting), methodology (supporting), validation (supporting), visualization (equal), writing – review and editing (equal). **Krithika Layagala:** investigation (supporting), methodology (supporting), validation (supporting), writing – review and editing (equal). **Pierrick Harnay:** resources (equal), writing – review and editing (equal). **Hollie M. Putnam:** conceptualization (equal), data curation (equal), funding acquisition (equal), resources (equal), visualization (equal), writing – original draft (supporting), writing – review and editing (equal). **Adrienne M. S. Correa:** conceptualization (equal), funding acquisition (equal), project administration (lead), resources (lead), supervision (lead), validation (equal), visualization (supporting), writing – original draft (supporting), writing – review and editing (equal).

## Funding

This research was funded by U.S. National Science Foundation Grant awards OCE‐2533138 to A.M.S.C., OCE‐2348674 to H.M.P. and OCE‐1829867 to S.C.B. This research was also supported by OCE‐2224354 (and earlier awards) to the Mo'orea Coral Reef LTER as well as a generous gift from the Gordon and Betty Moore Foundation.

## Disclosure

Benefit‐Sharing Statement: The results of this research, and all associated data and protocols, are open access and are being shared with the provider communities and the broader scientific community. This research can support conservation and restoration activities related to the organisms being studied—a priority concern in the provider community.

## Conflicts of Interest

The authors declare no conflicts of interest.

## Supporting information


**Data S1:** ece374409‐sup‐0001‐DataS1.txt.

**Data S2:** ece374409‐sup‐0002‐DataS2.txt.

**Data S3:** ece374409‐sup‐0003‐DataS3.txt.

**Figure S1:** ece374409‐sup‐0004‐supinfo01.pdf.

## Data Availability

All sequence data required for reproduction of these analyses are available on public databases or have been provided as supplemental data, as documented in the main text of the manuscript. A Sanger sequence representing new genetic diversity of the PocHistone gene has been deposited in GenBank under accession number PZ381813. An example protocol with suggested materials and methods for executing this workflow is published (DOI: 10.17504/protocols.io.bp2l6j57rvqe/v1).
