# Supplementary material for: A Genetic Method for Distinguishing Cryptic Pocillopora Species in French Polynesia Without Sequencing
Source: Ecol Evol. 2026 Oct 2;16(10):e74409. doi: 10.1002/ece3.74409 (PMC13631713; doi:10.1002/ece3.74409)
Supplement: Supplementary file 1 [file ECE3-16-e74409-s002.pdf]

Supplemental Materials and Methods for:

A Genetic Method for Distinguishing Cryptic *Pocillopora* Species in French Polynesia without Sequencing

Francesca M. Cohn[1,2], Erika Johnston[3], Scott Burgess[4], Jordan A. Sims[1], Krithika Layagala[1], Pierrick Harnay[5], Hollie M. Putnam[6], Adrienne M.S. Correa[1]

[1]Department of Environmental Science, Policy, and Management, University of California, Berkeley, CA, 94720-7000 USA

[2]Stanford University, Department of Developmental Biology, 450 Jane Stanford Way, Stanford, CA 94305-2004, USA.

[3]University of Hawaii at Manoa, Hawaii Institute of Marine Biology, Kaneohe, HI, 96744-3617 USA

[4]Florida State University, Department of Biological Science, 319 Stadium Drive, Tallahassee, FL, 32306-4296, USA.

[5]University of Haifa, Leon H. Charney School of Marine Sciences, Haifa, Haifa District, 3103301 Israel

[6]University of Rhode Island, College of the Environment and Life Sciences, Department of Biological Sciences, Kingston, RI 02881-2003 USA

In Vitro *Uniformed Validation*

Pocillopora corals were sampled from 2m depth in the lagoon at four separate sites on the north shore of the island of Moorea between 2022-2023 under the French Polynesian Government Délégation à la Recherche (Protocole d'Accueil 2022–2023) and transported under CITES permit FR2398700018-E. Tissue biopsies were stored in DNA/RNA Shield reagent (Zymo Research #R1100-250) at -20°C until DNA extraction. DNAs were extracted using the Quick-DNA 96 Plus Kit (Zymo Research #D4070) following manufacturer's instructions with the addition of proteinase K. Species identification was completed at the University of Rhode Island (URI) following mtORF amplification and Sanger sequencing of the forward primer (FatP6.1; Flot et al., 2008) for *Pocillopora acuta*, *Pocillopora* cf. *effusa*, *Pocillopora tuahiniensis*, and *Pocillopora verrucosa*, followed by PocHistone amplification (PocHistoneF and PocHistoneR primers; Flot et al., 2008) and *XhoI* RFLP for *Pocillopora meandrina* and *Pocillopora grandis* according to Johnston et al. (2018). To confirm the identity of the 36 unknown *Pocillopora* samples used in the uninformed validation (**Supplemental Table S1**), a phylogenetic species tree was generated from their mtORF sequences as well as those of NCBI reference sequences of known haplotype identities **(Supplemental Figure S2).**

BanI/BceAI Digest

An additional co-digest with *BanI* and *BceAI* is available to differentiate *P.* cf. *effusa* from all other species; this digest groups *P. grandis* (Haplotype 1), *P. meandrina* (Haplotype 1a, 1c, 1d, 1e, 8a, 9), and *P. acuta* (Haplotype 5a) for differentiation with additional digests. *P.* cf. *effusa* shares a fixed guanine SNP at position 312 with *P. acuta*, *P. meandrina*, and *P. grandis*. However, *P. acuta* and *P. meandrina* share a guanine SNP at position 211 with all other species except for *P.* cf. *effusa*.

Five microliters of the mtORF amplicon of each species was digested with 0.5 ul of *BanI* and 0.5 ul of *BseAI* in 0.5ul of rCutSmart™ Buffer for 1 hour at 37°C followed by 20 min at 65°C. The product was then run out on a 2% agarose gel at 100 V for 45 min.

All successful digests in **Figure S4** were accurately identified in the uninformed validation using *BanI/BceAI* (substituted for *EcoRV-HF*). However, *BanI/BceAI* was not chosen for the ***Finalized Sequential Protocol*** because it requires two enzymes, instead of the one enzyme required when performing the *EcoRV-HF* digest.

RADseq Histone Analysis

To investigate why some *P. grandis* samples produced a triple banding pattern following digestion of the PocHistone region with the *XhoI* restriction enzyme, and to determine the phylogenetic placement of the mtORF haplotype 9, we generated an identity-by-state matrix using restriction site-associated DNA sequencing (RADseq) libraries and investigated bam mapping alignments of the PocHistone region using those same libraries (Burgess and Johnston, unpublished data). Samples (<1 cm) were collected from 384 *Pocillopora* corals from five species (*P.* cf. *effusa, P. grandis*, *P. meandrina*, *P. tuahiniensis*, and *P. verrucosa*) at 5, 10, and 20 m depths from the fore reefs of Mo'orea in 2019 and 2021. Genomic DNA was extracted from these samples using Omega BIO-TEK E-Z 96 Tissue DNA Kits (D1196-01). Two elutions (50 and 100 ul) were collected in HPLC water and combined. Samples were identified to species prior to RADseq by sequencing the mtORF region and then using the histone RFLP assay to differentiate *P. meandrina* and *P. grandis* following Johnston et al. (2018).

RADseq libraries were prepared by digesting genomic DNA with the *DpnII* restriction enzyme (New England Biolabs: R0543S). Half reactions of the KAPA HyperPrep Kit (Roche: KK8504) and xGen™ Stubby Adapter and UDI Primers Pairs (Integrated DNA Technologies: 10005921) were used to prepare libraries. DNA fragment size selection was carried out post adapter ligation, pre library amplification, using Mag-Bind® TotalPure NGS beads (Omega BIO-TEK: M1378-01). Equimolar amounts of each library were then pooled and sequenced across four lanes of the HiSeq platform (2 x 150bp).

To identify the placement of mtORF haplotype 9, demultiplexed reads were first mapped to the *P. meandrina* genome (Stephens et al., 2022) using BWA (Li and Durbin, 2009) and bam files were filtered with SAMtools (Li et al., 2009) to remove reads with a mapping quality score below 20 and those with secondary alignments (samtools view -b -q 20 -F 0x0100). Genotype likelihoods for each sample were estimated using ANGSD (v0.940; "-domajorminor 1, -gl 2, -domaf 2, -docounts 1, -dopost 1, -dogeno 16, -doGlf 2, -SNP_pval 1e-7, -minInd 310, -nInd 387, -minmapQ 30, -minQ 30, -doBcf 1, -geno_minDepth 10, -dumpCounts 2, -doIBS 1, -doCov 1, -makeMatrix 1") (Korneliussen et al., 2014) and the resulting identity-by-state matrix was visualized to identify *Pocillopora* species placement. All haplotype 9 samples grouped with *P. meandrina*.

To investigate why a triple banding pattern, instead of the expected double banding pattern, was produced from PocHistone digestion with the *XhoI* restriction enzyme, RADseq libraries from the nine libraries identified as *P. grandis* in the identity-by-state matrix were mapped to the PocHistone region (GenBank accession: MG587096; Johnston et al. (2018)) using BWA-MEM (Li, 2013). One sample that had been identified as *P. meandrina* by the combination of mtORF sequencing and histone RFLP assay grouped with the *P. grandis* clade in the identity-by-state matrix. RADseq data from this library were also mapped to the PocHistone region. The PocHistone bam files were then visually inspected in Geneious Prime 2025.1.1. The two libraries that produced the expected double banding pattern for *P. grandis* were found to be homozygous for the restriction enzyme cut site used to identify *P. grandis* (C'TCGAG). The six libraries that produced a triple banding pattern for the restriction enzyme cut site used to identify *P. grandis* displayed heterozygosity at the cut site in the bam files (CTCGAG / CTCGCG). The one library that was identified as *P. meandrina* using the RFLP assay, but which grouped with *P. grandis* in the identity-by-state matrix, was found to be homozygous for the PocHistone site used to identify *P. meandrina* (CTCGCG).

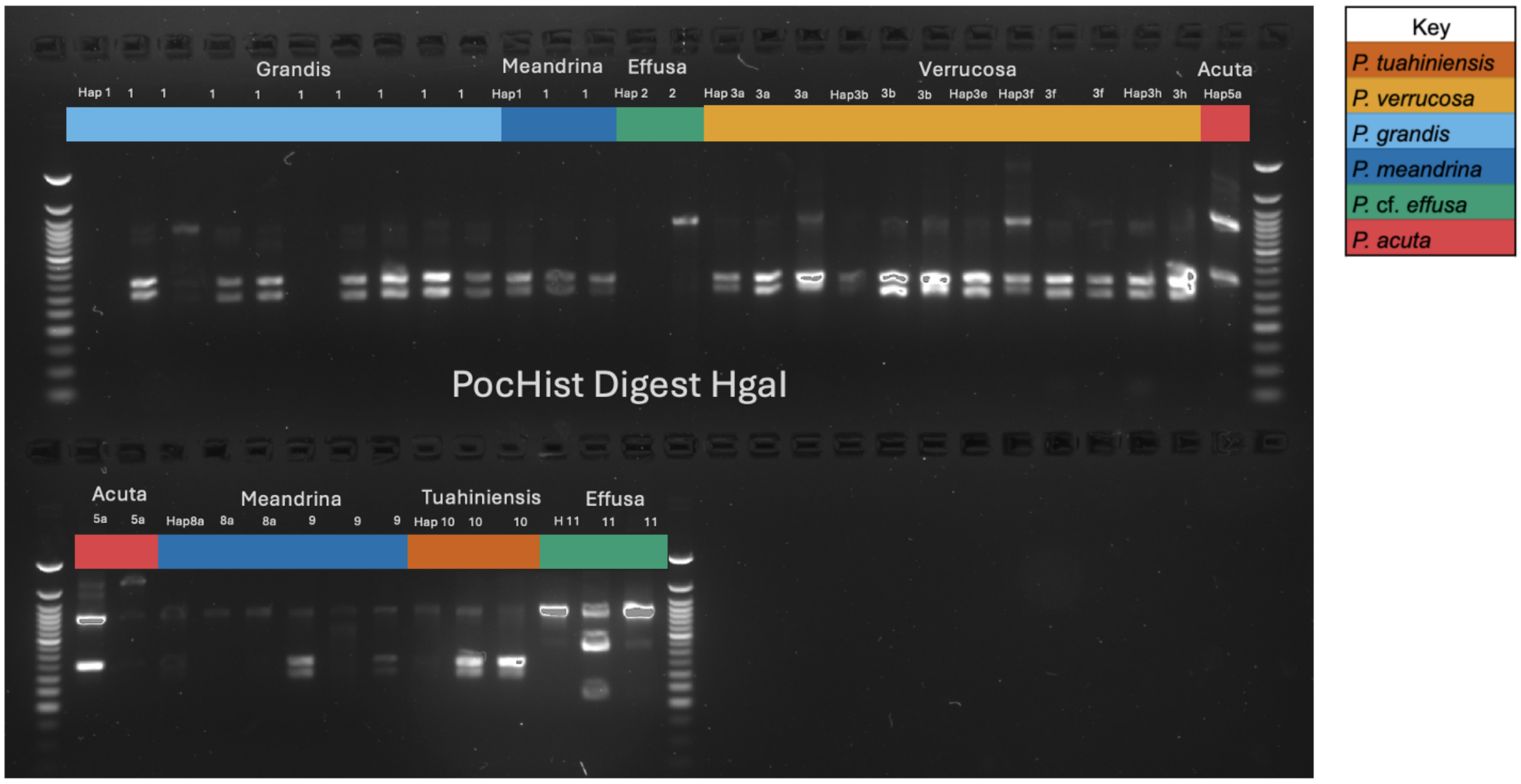

Figure S1. Results from an example *in vitro* test of candidate restriction enzymes on the PocHistone amplicon. The attempted *HgaI* digest was run (respectively) on PocHistone amplicons of 42 known *Pocillopora* species representing 12 haplotypes collected in French Polynesia and was designed to differentiate *P.* cf. *effusa* and *P. acuta* from all other species. Digests in lanes 1, 6, and 14 were unsuccessful on this gel. Variability of within-species digest pattern (i.e., *P.* cf. *effusa*) suggested that this was not a good candidate enzyme to use and further demonstrated the difficulties of using the PocHistone amplicon to differentiate species beyond *P. meandrina* and *P. grandis*.

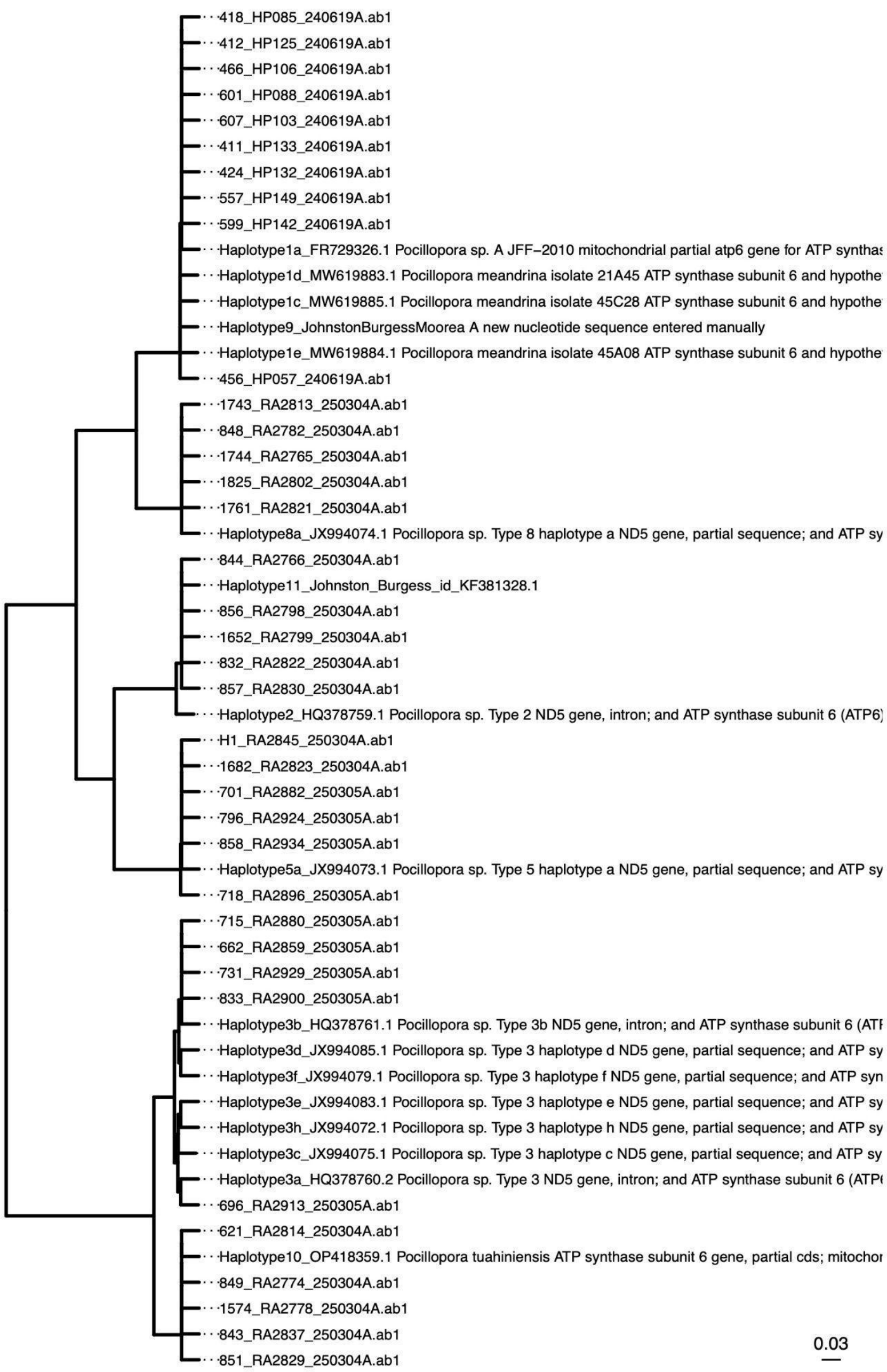

Figure S2. Species tree containing GenBank reference sequences of known *Pocillopora* haplotype identities (accession numbers in sequence name), along with sequences of the 36 unknown *Pocillopora* samples (samples ending in ".abi") used in the ***In Silico and In Vitro Uninformed Validation of the Protocol*** **(Supplemental Data S3)**. PocHistone amplicons for these samples were identified prior to the uninformed validation by *XhoI* digest.

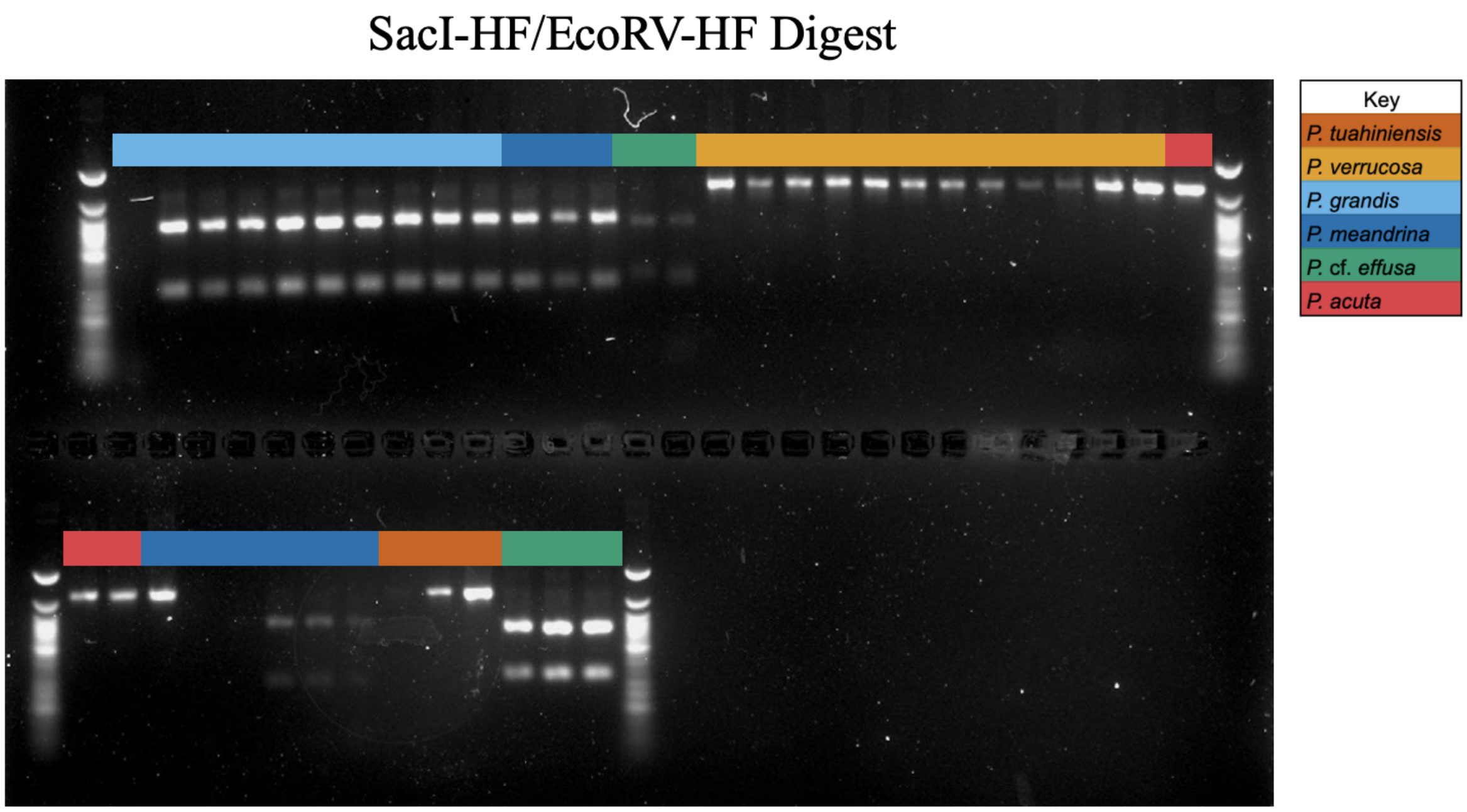

Figure S3. Results from initial *in vitro* test of candidate restriction enzymes. The attempted *SacI-HF/EcoRV-HF* digest was run (respectively) on the MtORF amplicon of 42 known *Pocillopora* species representing 12 haplotypes (***In Vitro Enzyme Testing Methods***) collected in French Polynesia. Digests in Lanes 1, 32, and 33 were unsuccessful on this gel. This digest was designed to differentiate *P.* cf. *effusa* from all other species while grouping *P. meandrina* with *P. grandis*, and *P verrucosa* with *P. tuahiniensis.* However, *SacI-HF* did not cut haplotype 8a of *P. meandrina*, meaning that haplotype 8a grouped with *P. verrucosa* and *P. acuta* rather than the other *P. meandrina* samples. Therefore, the *SacI-HF/EcoRV-HF* digest was not selected for the ***Finalized Sequential Protocol*** because not all haplotypes of the same *Pocillopora* species produced the same digestion pattern.

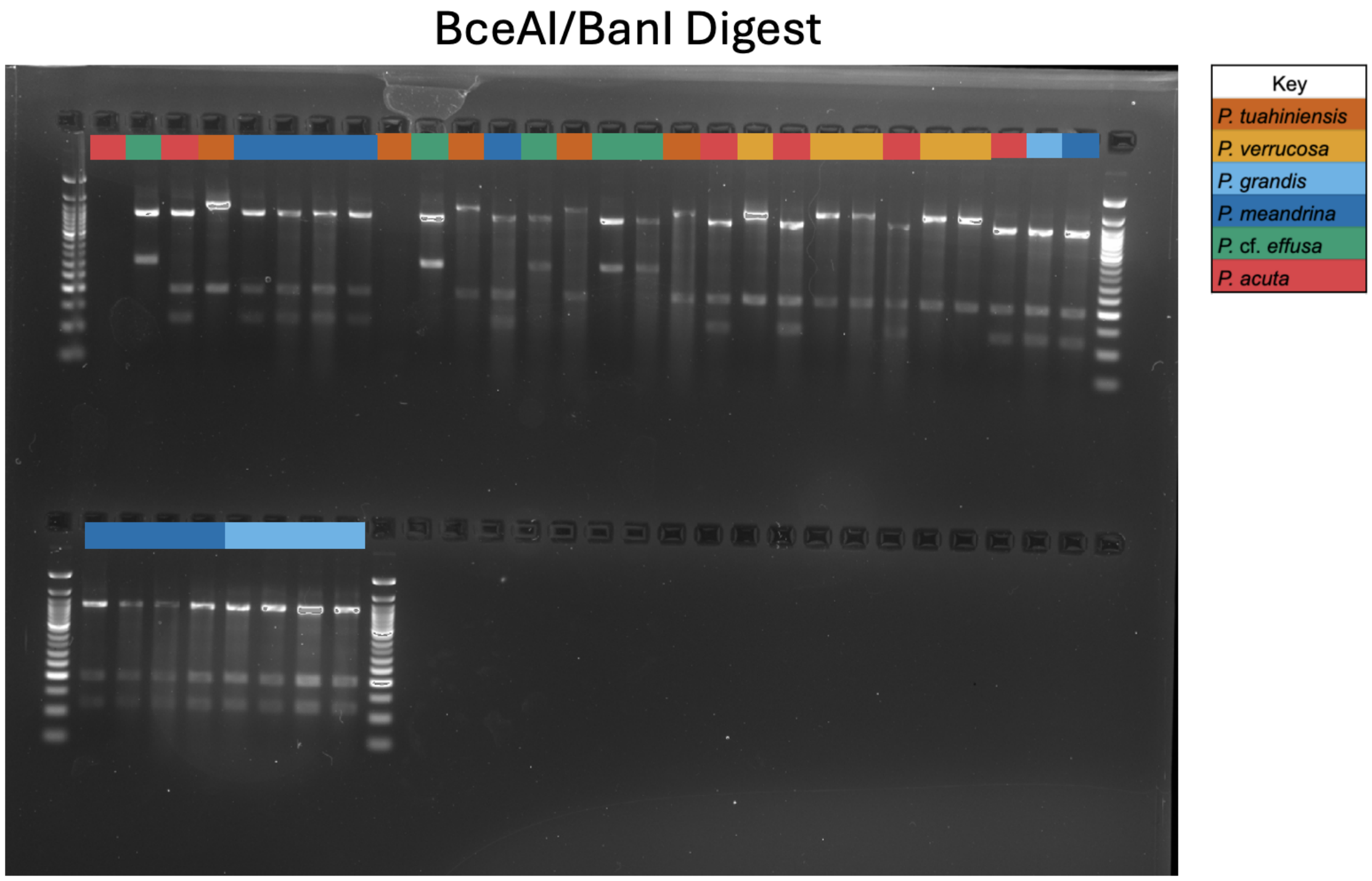

Figure S4. Results of the *in vitro uninformed validation* for the *BceAI/BanI* digest run on 36 unidentified *Pocillopora* samples representing *P. acuta* (n = 6 haplotype 5a), *P. meandrina* (n = 10 haplotypes 1a; 8a), *P.* cf. *effusa* (n = 5 haplotype 11), *P. grandis* (n = 5 haplotype 1a), *P. tuahiniensis* (n = 5 haplotype 10), and *P. verrucosa* (n = 1 haplotype 3a, n = 4 haplotype 3b) collected in French Polynesia. Digests in lanes 1 and 9 were unsuccessful on this gel. *BanI* and *BceAI* correctly differentiated *P.* cf. *effusa* from all other species, and grouped *P. grandis* (Haplotype 1), *P. meandrina* (Haplotype 1a, 1c, 1d, 1e, 8a, 9), and *P. acuta* (Haplotype 5a). However, *BceAI/BanI* digests were not included in the ***Finalized Sequential Protocol*** because *EcoRV-HF* was able to differentiate *P.* cf. *effusa* with only one enzyme**.**

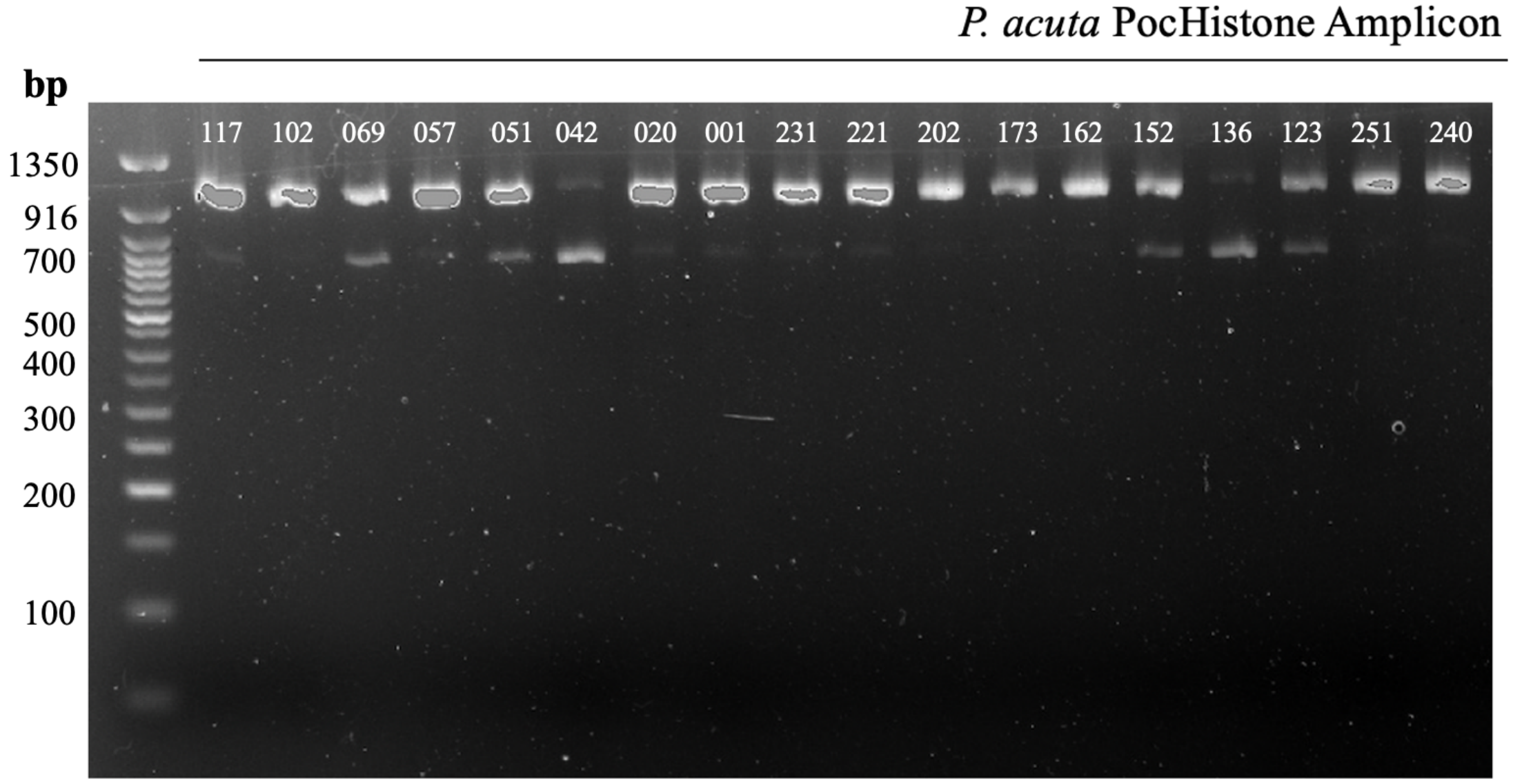

Figure S5. Undigested *Pocillopora acuta* PocHistone amplicon showing bands at 1,014 bp and 669 bp (or both), depending on whether the sample is likely homozygous or heterozygous for the 345 bp indel in the PocHistone amplicon. Samples from the collection of S. Burgess.

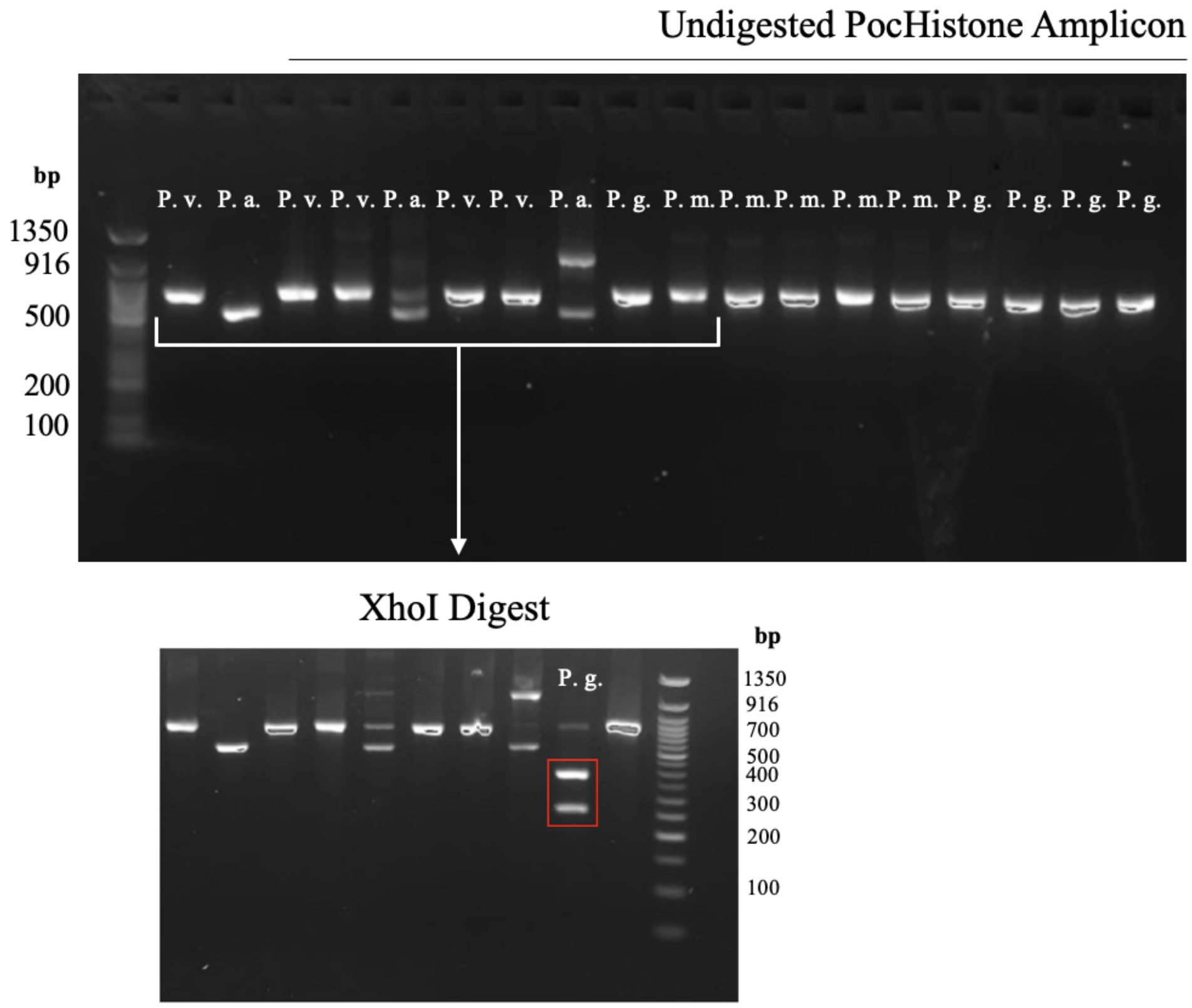

Figure S6. Top gel: Undigested PocHistone amplicon for *Pocillopora verrucosa* (P.v.), *Pocillopora acuta* (P.a.), *Pocillopora grandis* (P.g.), and *Pocillopora meandrina* (P.m.). Different band size anomalies are noticeable in the second, fifth, and eight wells - all *P. acuta* samples. Bottom gel: Several of the PocHistone amplicons above (white bracket and arrow) are digested with *XhoI*. Despite the anomalies present in the *P. acuta* bands, the *P. grandis* sample (well 9) is the only sample showing the characteristic band sizes of 287 bp and 382 bp that identify it (red box).

**Date.Collected. dd.mm.yy**	**longitude**	**latitude**	**Sample. ID**	**Species**
9/4/22	NA	NA	H1	*P. acuta*
23/03/23	-149.8526452	-17.48223933	1652	*P.* cf. *effusa*
23/03/23	-149.8526452	-17.48223933	1682	*P. acuta*
23/03/23	-149.8526452	-17.48223933	1574	*P. tuahiniensis*
27/03/23	-149.8526452	-17.48223933	1825	*P. meandrina*
27/03/23	-149.8526452	-17.48223933	1744	*P. meandrina*
27/03/23	-149.8526452	-17.48223933	1743	*P. meandrina*
27/03/23	-149.8526452	-17.48223933	1761	*P. meandrina*
5/2/23	-149.8344367	-17.47810177	843	*P. tuahiniensis*
5/2/23	-149.8344367	-17.47810177	844	*P.* cf. *effusa*
5/2/23	-149.8344367	-17.47810177	849	*P. tuahiniensis*
5/2/23	-149.8344367	-17.47810177	848	*P. meandrina*
5/2/23	-149.8344367	-17.47810177	856	*P.* cf. *effusa*
5/2/23	-149.8344367	-17.47810177	621	*P. tuahiniensis*
5/2/23	-149.8344367	-17.47810177	832	*P.* cf. *effusa*
5/2/23	-149.8344367	-17.47810177	857	*P.* cf. *effusa*

5/2/23	-149.8344367	-17.47810177	851	*P. tuahiniensis*
5/2/23	-149.8344367	-17.47810177	858	*P. acuta*
4/2/23	-149.8344367	-17.47810177	715	*P. verrucosa*
4/2/23	-149.8344367	-17.47810177	718	*P. acuta*
4/2/23	-149.8344367	-17.47810177	696	*P. verrucosa*
4/2/23	-149.8344367	-17.47810177	731	*P. verrucosa*
4/2/23	-149.8344367	-17.47810177	701	*P. acuta*
4/2/23	-149.8344367	-17.47810177	662	*P. verrucosa*
7/2/23	-149.8076461	-17.47569278	833	*P. verrucosa*
7/2/23	-149.7835691	-17.47304608	796	*P. acuta*
5/10/22	-149.8076461	-17.47569278	456	*P. grandis*
6/10/22	-149.8076461	-17.47569278	466	*P. meandrina*
5/10/22	-149.8076461	-17.47569278	424	*P. meandrina*
5/10/22	-149.8076461	-17.47569278	418	*P. meandrina*
5/10/22	-149.8076461	-17.47569278	412	*P. meandrina*
5/10/22	-149.8076461	-17.47569278	411	*P. meandrina*
6/10/22	-149.8076461	-17.47569278	557	*P. grandis*
5/10/22	-149.8076461	-17.47569278	599	*P. grandis*

6/10/22	-149.8076461	-17.47569278	607	*P. grandis*
6/10/22	-149.8076461	-17.47569278	601	*P. grandis*

Table S1. Collection and identification information for 36 *Pocillopora* samples (Putnam Lab, URI) used in the uninformed *in vitro* identification.

Focal *Pocillopora* Species	Haplotype IDs	Amplicon	Restriction Enzyme	Resulting Fragment Size(s) for Focal Species (bp)	Resulting Fragment Size(s) for Non-Focal Species (bp)	Additional mtORF Digests Required to ID Focal Species?	Additional Amplicon?	Restriction Enzyme	Resulting Fragment Size(s) for Focal Species (bp)	Resulting Fragment Sizes for Non-Focal Species (bp)
P. verrucosa	3a, 3b, 3c, 3d, 3e, 3f, 3h	mtORF	BseYI C'CCAGC GGGTC'G AciI C'CGC GGC'G	209, 337, 432	*P. tuahiniensis:* 149, 188, 209, 432; All other species: 432, 546	No	No	N/a	N/a	N/a
P. tuahiniensis	10	mtORF	BseYI C'CCAGC GGGTC'G AciI C'CGC GGC'G	149, 188, 209, 432	*P. verrucosa*: 209, 337, 432; All other species: 432, 546	No	No	N/a	N/a	N/a
P. cf. *effusa*	2, 11	mtORF	BceAI ACGGC(N)12' TGCCG(N)14' BanI G'GYRCC CCRYG'G	311, 667	*P. acuta, P. grandis, P. meandrina*: 115, 196, 667; *P. tuahiniensis, P. verrucosa*: 196, 782	No	No	N/a	N/a	N/a
P. acuta	5a	mtORF	NlaIV GGN'NCC CCN'NGG	30, 172, 313, 463	*P.* cf. *effusa*, *P. grandis*, *P. meandrina*: 202, 313, 463; *P. tuahiniensis*, *P. verrucosa*: 463, 515	No	No	N/a	N/a	N/a

P. meandrina	1a, 1c, 1d, 1e, 8a, 9	mtORF	BceAI ACGGC(N)12’ TGCCG(N)14’ BanI G’GYRCC CCRYG’G	115, 196, 667	*P. acuta*, *P. grandis*: 115, 196, 667; *P. tuahiniensis*, *P. verrucosa*: 196, 782; *P.* cf. *effusa*: 311, 667	NlaIV to distinguish from *P. acuta*	PocHistone	XhoI C’TCGAG GAGCT’C	669 (Johnston et al., 2018)	*P. grandis*: 287, 382; all other species: 669
P. grandis	1a	mtORF	BceAI ACGGC(N)12’ TGCCG(N)14’ BanI G’GYRCC CCRYG’G	115, 196, 667	*P. acuta*, *P. grandis*: 115, 196, 667; *P. tuahiniensis*, *P. verrucosa*: 196, 782; *P.* cf. *effusa*: 311, 667	NlaIV to distinguish from *P. acuta*	PocHistone	XhoI C’TCGAG GAGCT’C	287, 382 (Johnston et al., 2018)	All other species: 669

Table S2. Summary of an alternative identification scheme for *Pocillopora* species in French Polynesia that uses a *BceAI/BanI* digest (rather than an *EcoRV-HF* digest). The table lists the haplotype ID for each *Pocillopora* species, the amplicon(s) and restriction enzyme(s) for identification, the resulting band fragment sizes for the focal species, and the resulting fragment size(s) for non-focal species when cut with that restriction enzyme. Corresponding information regarding additional digests required (if any) to fully identify each *Pocillopora* species are provided. Note that the 30 bp fragment resulting from cutting MtORF amplicons of *P. acuta* with *NlaIV* is too small to be reliably observed on a 2% agarose gel; instead a band pattern of 172, 313, and 463 bp fragments should be visible for this species.

References

Flot, J.-F., Magalon, H., Cruaud, C., Couloux, A., & Tillier, S. (2008). Patterns of genetic structure among Hawaiian corals of the genus *Pocillopora* yield clusters of individuals that are compatible with morphology. *Comptes Rendus Biologies*, *331*(3), 239–247. https://doi.org/10.1016/j.crvi.2007.12.003

Johnston, E. C., Forsman, Z. H., & Toonen, R. J. (2018). A simple molecular technique for distinguishing species reveals frequent misidentification of Hawaiian corals in the genus *Pocillopora*. *PeerJ*, *6*, e4355. https://doi.org/10.7717/peerj.4355

Korneliussen, T. S., Albrechtsen, A., & Nielsen, R. (2014). ANGSD: Analysis of Next Generation Sequencing Data. *BMC Bioinformatics*, *15*, 356. https://doi.org/10.1186/s12859-014-0356-4

Li, H. (2013). Aligning sequence reads, clone sequences and assembly contigs with BWA-MEM. *arXiv*. https://doi.org/10.48550/arXiv.1303.3997

Li, H., & Durbin, R. (2009). Fast and accurate short read alignment with Burrows–Wheeler transform. *Bioinformatics*, *25*(14), 1754–1760. https://doi.org/10.1093/bioinformatics/btp324

Li, H., Handsaker, B., Wysoker, A., Fennell, T., Ruan, J., Homer, N., Marth, G., Abecasis, G., Durbin, R., & 1000 Genome Project Data Processing Subgroup. (2009). The Sequence Alignment/Map format and SAMtools. *Bioinformatics*, *25*(16), 2078–2079. https://doi.org/10.1093/bioinformatics/btp352

Stephens, T. G., Lee, J., Jeong, Y., Yoon, H. S., Putnam, H. M., Majerová, E., & Bhattacharya, D. (2022). High-quality genome assemblies from key Hawaiian coral species. *GigaScience*, *11*, giac098. https://doi.org/10.1093/gigascience/giac098
